# Depression in Parkinson's disease: A case-control study

**DOI:** 10.1371/journal.pone.0192050

**Published:** 2018-02-01

**Authors:** Yu-Hsuan Wu, Yi-Huei Chen, Ming-Hong Chang, Ching-Heng Lin

**Affiliations:** 1 Section of Neurology, Taichung Veterans General Hospital, Taichung, Taiwan; 2 Department of Medical Education and Research, Taichung Veterans General Hospital, Taichung, Taiwan; 3 Department of Neurology, National Yang-Ming University School of Medicine, Taipei, Taiwan; Oslo Universitetssykehus, NORWAY

## Abstract

**Background:**

To evaluate the association between Parkinson’s disease (PD) prognosis and the patient’s onset of depression.

**Methods:**

A total of 353 patients with newly-diagnosed PD and a history of depression were enrolled. On the basis of the onset of depression before or after PD diagnosis, we divided participants into PD patients with pre- or post-diagnostic depression. Cox’s regression analysis was used to detect risks between the onset of depression and outcomes (including death, accidental injury, dementia, and aspiration pneumonia). The association between the onset of depression and levodopa equivalent dosage (LED) and cumulative equivalent dosage of antidepressants were assessed.

**Results:**

PD patients with post-diagnostic depression were associated with significantly higher risks of dementia (adjusted HR = 2·01, *p* = 0·015), and were older (58·5 ± 17·7 vs. 53·7 ± 18·6, *p* = 0·020) at the time of PD diagnosis than PD patients with pre-diagnostic depression. The higher incident rate of accidental injury was also noted in PD patients with post-diagnostic depression (48·1 vs. 31·3/1000 person-years, HR = 1·60, *p* = 0·041), but no statistical significance was observed in the adjusted hazard ratio (HR) (HR = 1·52, *p* = 0·069). Otherwise, mortality, motor condition and severity of depression revealed no significant difference between PD patients with pre-diagnostic and post-diagnostic depression.

**Conclusion:**

PD patients with post-diagnostic depression had higher incidence of dementia, implying different onset time of depression could be associated with different subtypes and spreading routes which should be examined in follow-up studies.

## Introduction

Parkinson’s disease (PD) is currently considered to be a highly heterogeneous, multisystem disorder with motor and nonmotor symptoms (NMS). Many recently published articles have described NMS-dominant phenotypes such as cognitive impairment, apathy, depression or anxiety, sleep disorder, olfactory loss, and dysautonomia. These reported NMS-dominated subtypes are consistent with the neuropathological studies, that suggesting differential pathways of neuronal degeneration and Lewy body deposition in the nondopaminergic brainstem and limbic areas of PD [1]. Moreover, NMS subtypes reflect the difference in spread of pathology as proposed by Braak et al.[2], Jellinger et al.[3], and Beach et al.[4].

According to the University of Virginia Movement Disorders Database, full autopsies were performed in ten PD patients. The majority of patients died of PD-related causes (six of ten patients). Five patients died of bronchopneumonia, with one patient dying of complications related to a hip fracture [5].The independent predictors of mortality included higher age, male sex, severity of motor symptoms, gait dysfunction, dementia, the presence of psychotic symptoms and depression [6].

Depression is the most common neuropsychiatric symptom in PD patients. Several studies have demonstrated an association between depression and cognitive impairments in PD patients. However a few other studies did not detect a significant association [7]. Depression might occur in the premotor or motor stage of PD. Our previous study revealed that PD patients with premotor symptoms (including depression, rapid eye movement sleep behavior disorder, and constipation) exhibited increased mortality and morbidity (dementia and aspiration pneumonia) compared to PD patients without premotor symptoms [8]. We hypothesized that the different onset times of depression before or after the diagnosis of PD could also affect PD prognosis. Therefore, the present study evaluated the association between the onset of depression from PD diagnosis and the outcomes of PD.

## Methods

### Study design and data sources

This study used data from Taiwan’s National Health Insurance Research Database (NHIRD), which were released by Taiwan’s National Health Research Insurance (NHRI). The NHRI program was instituted in 1995, and by 2010 it covered nearly 98% of all residents in Taiwan [9].This cohort dataset comprises all comprehensive medical care coverage between 1996 and 2011 of a randomly-selected group of beneficiaries (n = 1 million) in the 2005 Registry of Beneficiaries. Diseases are coded according to the International Classification of Disease, 9^th^ revision, Clinical Modification (ICD-9-CM) Diagnosis Codes, 2001 edition. The release of data was approved by the Ethics Committee of NHRI, as the study protocol complied with the Declaration of Helsinki. The distribution of study subjects was representative of the national population in Taiwan. In addition, because participation in NHI is mandatory and easily accessible with low payments, follow-up compliance is high.

### Study population

Patients were defined as newly-diagnosed PD patients if they received an ICD-9-CM code of 332·0 [9] between January 2001 and December 2008. In Taiwan, most patients (82·9%) received their first anti-PD medication in referred hospitals. About half of PD patients received their initial prescription from neurologists [10]. A previous validation study using a hospital administrative database reported a positive predictive value of more than 90% by using this definition of PD [11].To further improve diagnostic validity, only patients whose diagnostic code of PD was made more than three times in outpatient services, or more than once during hospitalization, were included. We excluded patients who were exposed to medicines with a high risk of drug-induced parkinsonism (Table A in S1 File) more than three times within three months before the diagnosis of PD. We excluded patients with the disease, potentially causing secondary or atypical Parkinsonism (Table B in S1 File). Participants who had never taken any anti-parkinsonian medicine after diagnosis of PD were also excluded. The definition of depressive disorder included major depressive disorder (ICD-9-CM codes: 292·2 and 296·3), dysthymic disorder (ICD-9-CM code: 300·4,309·0,309·1) and depressive disorder not elsewhere classified (ICD-9-CM code: 311) [12] occurring at least three or more times at outpatient clinics, or more than once during hospitalization. PD patients with depression were included in the final study group.

### Pre-diagnostic depression vs. Post-diagnostic depression

The study subjects were divided into two subgroups on the basis of depression onset before or after the PD diagnosis. The codes for pre-diagnostic depression were extracted in the years before diagnosis of PD; patients with pre- and post-diagnostic depression were observed until death or December 2011.

### Confounders

Potential confounders included hypertension, diabetes mellitus (DM), hyperlipidemia, and ischemic heart disease (IHD) (Table C in S1 File). IHD was selected due to the cause of mortality in PD patients. Hypertension, DM, and hyperlipidemia were selected because they are risk factors for cardiovascular disease.

### Outcomes

The primary clinical outcome was mortality. Other associated prognostic factors were accidental injury, dementia, and aspiration pneumonia (Table D in S1 File). The secondary clinical outcome was levodopa equivalent dosage (LED) of all anti-parkinsonian drugs at the end of every year after diagnosis of PD and antidepressant use. Antidepressants were classified into 4 groups according to their proposed mechanisms [12]: tricyclic antidepressants (TCA), selective serotonin reuptake inhibitors (SSRIs), monoamine oxidase inhibitors (MAOIs), and other antidepressants. The severity of depression was based on the 1-year-cumulative equivalent dosage of antidepressants (fluoxetine = 1) in the last observational year (Table E in S1 File).

### Statistics

Stata version 11·1 (College Station, TX, USA) was used to perform all statistical analyses. Student’s t test and Pearson chi-squared test were used to compare age, distribution of gender, and confounders (hypertension, DM, hyperlipidemia, and IHD) between the two groups of PD patients.

The crude person-time incidence rate (per 1000) was calculated as the number of incident cases divided by the combined person-years from each subject in the cohort. A comparison of risk of death, accidental injury, dementia, and aspiration pneumonia between PD patients with pre-and post-diagnostic depression were presented as crude HRs. The adjusted HRs were estimated in a multivariate Cox proportional hazards model adjusting for age, gender, hypertension, DM, hyperlipidemia, and IHD.

To clarify the impact of confounders, Cox models were also calculated separately for each of the following factors: hypertension, DM, hyperlipidemia, and IHD.

We also used the Student’s t-test to analyze LED at the end of every year after diagnosis of PD and the 1-year-cumulative equivalent dosage of antidepressants at the last observational year (in subjects with pre- and post-diagnostic depression). P values <0·05 or CIs for HRs that excluded the value 1·00 were statistically significant.

## Results

### Baseline cohort characteristics

Table 1 shows the demographic characteristics and comorbidities of study participants. A total of 1213 patients diagnosed with PD from January 2001 to December 2008 were selected. Of these, 353 PD patients received a diagnosis of depression during or before the PD disease course. Among PD patients with depression, 41·9% were male, while the average age at the time of PD diagnosis was 55·4 ± 18·4 years. Patients with pre-diagnostic depression (*n* = 227) accounted for 64·3% of the PD patients with depression. PD patients with post-diagnostic depression was significantly older than patients with pre-diagnostic depression (58·5 ± 17·7 vs. 53·7 ± 18·6, *p* = 0·020). The interval between the PD diagnosis and dementia showed no clinical significance between PD with post-diagnostic depression and pre-diagnostic depression (6·5±3·0 years vs. 6·3±2·7, *p* = 0·538). No difference in the distribution of gender and comorbidities was present. The total mean follow-up time was 6·97 ± 2·43 years, and the mean follow-up time was longer in patients with post-diagnostic depression than those with pre-diagnostic depression (7·52 ± 2·24 years vs. 6·66 ± 2·47 years).

**Table 1:** Clinical characteristics of PD patients with pre-diagnostic and post-diagnostic depression.

Variable	Total (N = 353)	Pre-diagnostic depression (N = 227)	Post-diagnostic depression (N = 126)	p-value
	N (%)	N (%)	N (%)	
Age, years (mean±SD) at time of PD diagnosis	55·4±18·4	53·7±18·6	58·5±17·7	0·020 ^a^
Disease duration (from diagnosis of PD to dementia)	6·4±2·8	6·3±2·7	6·5±3·0	0·538 ^a^
Gender (male)	148 (41·9)	93(41·0)	55(43·7)	0·625
Hypertension ^b^	106 (30·0)	68(30·0)	38(30·2)	0·968
Diabetes ^c^	48 (13·6)	31(13·7)	17(13·5)	0·966
Hyperlipidemia ^d^	47(13·3)	30(13·2)	17(13·5)	0·942
Ischemic heart disease ^e^	44 (12·5)	26(11·5)	18(14·3)	0·440

^a^ Student’s t test; chi-squared test for all other p-values.

^b^ Definition of hypertension: patients with diseases of the following ICD codes: 401–405 / A260,A269.

^c^ Definition of diabetes: patients with diseases of the following ICD codes: 250/ A181.

^d^ Definition of hyperlipidemia: patients with diseases of the following ICD codes: 272/ A182.

^e^ Definition of ischemic heart disease: patients with diseases of the following ICD codes: 410–414.

Comorbidities was defined as at least once clinical diagnosis within 1 year before PD first diagnosis date

PD: Parkinson’s disease, N: case number, %: percentage. SD: standard deviation

### Hazard ratios of outcome variables associated with onset of depression

The outcome results between patients with post-diagnostic depression and pre-diagnostic depression in terms of death, dementia, accidental injury, and aspiration pneumonia are summarized in Table 2. The incident rate of death revealed no significant difference between PD patients with post-diagnostic depression and pre-diagnostic depression (23·2/1000 person-years vs. 26·5/1000 person-years, *p* = 0·471). After adjusting for age, sex, and comorbidities, the HR for mortality still demonstrated no statistical significance (adjusted HR = 0·68, *p* = 0·146). Compared with the pre-diagnostic depression, the incidence rates of accidental injury, aspiration pneumonia, and dementia were relatively higher in patients with post-diagnostic depression (48·1 vs. 31·3; 8·6 vs. 5·3; 34·2 vs. 15·4/1000 person-years, respectively). Except for aspiration pneumonia (HR = 1·50, *p* = 0·417), the crude HRs of accidental injury and dementia revealed statistical significance (HR = 1·60; 2·28, *p* = 0·041; 0·004, respectively). However, after adjusting for sex, age, and comorbidities, the HR demonstrated statistical significance only for dementia (HR = 1·52, *p* = 0·069 for accidental injury; HR = 1·13, *p* = 0·807 for aspiration pneumonia; and HR = 2·01, *p* = 0·015 for dementia).

**Table 2:** Incidence and hazard ratio of outcome variables associated with pre-diagnostic and post-diagnostic depression in Cox's regression analysis.

Outcome	Pre-diagnostic depression			Post-diagnostic depression			Crude HR (p-value)	Adjusted HR(95% CI)	p-value
	(N = 227)			(N = 126)					
	Event	Person-years	Incident rate (per1000 person-years)	Event	Person-years	Incident rate (per1000 person-years)			
Death	40	1512	26·5	22	947	23·2	0·83 (0·471)	0·68 (0·40–1·14)	0·146
Accidental injury	40	1278	31·3	36	748	48·1	1·60 (0·041)	1·52 (0·97–2·40)	0·069
Aspiration pneumonia	8	1501	5·3	8	925	8·6	1·50 (0·417)	1·13 (0·42–3·06)	0·807
Dementia	22	1430	15·4	28	818	34·2	2·28 (0·004)	2·01 (1·14–3·53)	0·015

Adjusted HR was adjusted for Age, Gender, Hypertension, Diabetes, Hyperlipidemia, Ischemic heart disease.

HR: hazard ratio, CI: confidence interval, N: case number, %: percentage.

### Hazard ratios of outcome variables based on comorbidities

Table 3 presents information about the effects of pre- or post-diagnostic depression in subgroups (according to comorbidities) of the study population’s prognosis. Compared with the pre-diagnostic depression, the adjusted HRs for dementia were significantly higher in patients with post-diagnostic depression (HR = 2·50, *p* = 0·033 in hypertensive patients; HR = 2·09, *p* = 0·015 in nonhyperlipidemic patients; and HR = 2·04, p = 0·026 in patients without IHD). In a diabetes-stratified analysis, PD patients with pre-diagnostic depression exhibited significantly higher mortality than PD patients with post-diagnostic depression (adjusted HRs = 0·17, *p* = 0·034). Adjusted HRs for accidental injury demonstrated statistical significance in post-diagnostic depressed PD patients with IHD (HR = 4·15, *p* = 0·046).

**Table 3:** Hazard ratio associated with pre-diagnostic and post-diagnostic depression in Cox's regression analysis, as stratified by co-morbidities.

Outcomes		Crude HR(p-value)	Adjusted HR(p-value)	(95% CI)
**Hypertension**				
No	Death	0·82 (0·576)	0·57 (0·143)	(0·27–1·21)
	Accidental injury	1·72 (0·050)	1·66 (0·076)	(0·95–2·90)
	Aspiration pneumonia	2·15 (0·192)	1·48 (0·516)	(0·46–4·79)
	Dementia	2·20 (0·041)	1·46 (0·344)	(0·67–3·18)
Yes	Death	0·82 (0·606)	0·79 (0·561)	(0·35–1·77)
	Accidental injury	1·35 (0·475)	1·34 (0·502)	(0·57–3·11)
	Aspiration pneumonia	0·46 (0·499)	0·51 (0·568)	(0·05–5·10)
	Dementia	2·54 (0·027)	2·50 (0·033)	(1·08–5·82)
**Diabetes**				
No	Death	1·09 (0·765)	0·95 (0·872)	(0·53–1·72)
	Accidental injury	1·55 (0·085)	1·37 (0·226)	(0·82–2·28)
	Aspiration pneumonia	2·06 (0·180)	1·67 (0·353)	(0·57–4·93)
	Dementia	2·29 (0·010)	1·80 (0·075)	(0·94–3·46)
Yes	Death	0·23 (0·055)	0·17 (0·034)	(0·03–0·87)
	Accidental injury	1·94 (0·216)	2·22 (0·175)	(0·70–7·07)
	Aspiration pneumonia			
	Dementia	2·49 (0·132)	2·42 (0·192)	(0·64–9·09)
**Hyperlipidemia**				
No	Death	0·88 (0·635)	0·75 (0·284)	(0·44–1·28)
	Accidental injury	1·72 (0·032)	1·61 (0·061)	(0·98–2·66)
	Aspiration pneumonia	1·45 (0·463)	1·13 (0·807)	(0·42–3·06)
	Dementia	2·43 (0·003)	2·09 (0·015)	(1·15–3·80)
Yes	Death			
	Accidental injury	1·09 (0·883)	1·16 (0·809)	(0·34–3·94)
	Aspiration pneumonia			
	Dementia	1·62 (0·611)	1·35 (0·775)	(0·17–10·75)
**Ischemic heart disease**				
No	Death	0·90 (0·710)	0·72 (0·264)	(0·40–1·28)
	Accidental injury	1·43 (0·153)	1·35 (0·235)	(0·82–2·23)
	Aspiration pneumonia	1·53 (0·394)	1·13 (0·807)	(0·42–3·06)
	Dementia	2·56 (0·003)	2·04 (0·026)	(1·09–3·81)
Yes	Death	0·51 (0·316)	0·59 (0·493)	(0·13–2·63)
	Accidental injury	2·93 (0·087)	4·15 (0·046)	(1·03–16·79)
	Aspiration pneumonia			
	Dementia	1·21 (0·780)	1·45 (0·603)	(0·36–5·83)

Adjusted HR was adjusted for Age, Gender, Hypertension, Diabetes, Hyperlipidemia, Ischemic heart disease.

HR: hazard ratio, CI: confidence interval, %: percentage.

### Levodopa equivalent dosage (LED) and cumulative equivalent dosage of antidepressants in each group

Table 4 presents the results of LED in patients with pre-diagnostic and post-diagnostic depression in the ensuing years. A trend of higher LED is observed in patients with post-diagnostic depression (except at the end of third year), but the difference was not statistically significant.

**Table 4:** Levodopa equivalent dosage in PD patients with and without premotor symptoms.

Levodopa equivalent dosage (mg/day) (mean±SD)	Pre-diagnostic depression	Post-diagnostic depression	p-value
end of 1^st^ year (N = 115)	259·1±168·4	267·3±143·5	0·780
end of 2^nd^ year (N = 95)	305·2±205·0	316·9±207·7	0·784
end of 3^rd^ year (N = 83)	383·5±258·2	360·1±190·7	0·636
end of 4^th^ year (N = 85)	365·4±236·2	395·3±211·8	0·541
end of 5^th^ (N = 77)	450·6±243·0	481·4±448·7	0·699
end of 6-8^th^ year (N = 78)	423·5±303·4	451·3±287·1	0·687
end of 8-11^th^ year (N = 42)	425·5±375·3	472·5±305·8	0·670

Student’s t-test for all p-values.

PD: Parkinson’s disease, N: case number, SD: standard deviation

The distribution of antidepressant use was similar between the two groups in the last year of observation. The two most frequently used antidepressants were SSRIs and other antidepressants (30·2% vs. 28·6% in the post-diagnostic depression and 20·7% vs. 24·2% in the pre-diagnostic depression, respectively). Depression appeared to be more severe in patients with post-diagnostic depression, based on higher 1-year cumulative equivalent dosage of antidepressants (fluoxetine = 1) in the last observational year; however, the difference was not statistically significant (1904·5 ± 3273·4 vs. 1739·5 ± 3440·1/year; *p* = 0·661).

## Discussion

Two main findings were seen in the current study: 1) PD patients with post-diagnostic depression were associated with significantly higher risks of dementia and were also older at the time of PD diagnosis than PD patients with pre-diagnostic depression; 2) mortality, accidental injury, motor condition and severity of depression revealed no significant difference between PD patients with pre-diagnostic and post-diagnostic depression.

Cognitive deficits may occur as a form of global cognitive decline or as an impairment of specific cognitive domains in depressed PD patients. For example, some studies found higher depression scores to be negatively correlated with lower scores on the Mini Mental State Examination (MMSE) [7]. Deficits of specific cognitive domains mainly involved impairment of executive functions, attention and memory [7]. In our study, PD patients with post-diagnostic depression had a significantly higher occurrence of dementia. This could possibly be explained by different spreading routes and older age. Increasing age is a major risk factor for the development of dementia in PD patients; thus the time for dementia development decreases with increasing age at the onset of PD [13].

According to recent studies [1], the possible routes of PD pathology spreading were determined from pathophysiological studies. The proposed concept (based on nonmotor subtypes in PD) comprised the brainstem route, the olfactory-limbic route, and the neocortical route. Characteristics of the brainstem route were late-onset hyposmia, sleep-dominant phenotype (including excessive daytime sleep and rapid eye movement sleep behavior disorder), and dysautonomia (adrenergic problem, gastrointestinal tract, and genitourinary symptoms). The brainstem route was determined through Braak staging of Lewy pathology. The olfactory-limbic route was characterized by early-onset anosmia and a limbic phenotype (which is depression-dominant, fatigue-dominant, pain-dominant, and weight-loss dominant). The neocortical route was characterized by older age of onset and cognitive-dominant phenotypes (dementia, amnestic mild cognitive impairment, and falls with cognitive impairment and apathy). Our results were consistent with the aforementioned finding. PD patients with pre-diagnostic depression and those with post-diagnostic depression appeared to be involved in different developmental routes, which tend to result in different phenotypes. The pathology of pre-diagnostic depression was involved in the caudal raphe nuclei of the brainstem route (Stage II) [14,15]. Post-diagnostic depression appeared to involve the cortical area of the neocortical route. Therefore, in PD patients with post-diagnostic depression, a significantly higher occurrence of dementia may be explained by early involvement in the neocortical area. The present study may suggest that the different developmental routes of pathology resulted in different phenotypes and clinical outcomes.

The main advantage of the present study was the use of a nationwide dataset with a large sample size. However, several limitations of the study should be discussed. First, our cross-sectional design demonstrated only associations between the onset of depression and prognosis in PD patients rather than a causal relationship. Second, we could not obtain a clinical review of patient data from NHIRD. As such, we could not completely exclude patients with secondary parkinsonism or atypical parkinsonism, although we exerted great effort to exclude that possibility. Third, information on PD prognoses, such as the Hoehn and Yahr stage, causes of death, cognitive tests of dementia, severity of motor symptoms, and formal autonomic function tests are not available in the NHIRD. Even though, we could not present detailed reports due to ICD code weakness, the trends found in the present study may still provide clinical information. Further population-based prospective studies are needed to better investigate the association between onset of depression and PD prognosis.

## Supporting information

S1 File Table A. Drugs with high risk of extrapyramidal symptoms. Table B. Diseases with risk of secondary or atypical Parkinsonism. Table C. Potential confounders. Table D. Primary clinical outcomes. Table E. Dose equivalent of anti-depressants to fluoxetine (fluoxetine = 1).
(DOC)
