# Supplementary material for: Depression in Parkinson's disease: A case-control study
Source: PLoS One. 2018 Feb 1;13(2):e0192050. doi: 10.1371/journal.pone.0192050 (PMC5794149; doi:10.1371/journal.pone.0192050)
Supplement: S1 File — Table A. Drugs with high risk of extrapyramidal symptoms. Table B. Diseases with risk of secondary or atypical Parkinsonism. Table C. Potential confounders. Table D. Primary clinical outcomes. Table E. Dose equivalent of anti-depressants to fluoxetine (fluoxetine = 1). (DOC) [file pone.0192050.s001.doc]

**Table A. Drugs with high risk of extrapyramidal symptoms**

| **Drug category** | **Generic drugs** |
| --- | --- |
| Anti-emetics | Metoclopramide, Prochlorperazine |
| Neuroleptics | Haloperidol, Amisulpride, Flupentixol, Fluphenazine, Levomepromazine, Pimozide, Amisulpride, Thioridazine, Zuclopenthixol, Risperidone, Olanzapine, Aripiprazole |
| Calcium channel blocker | Flunarizine, Cinnarizine |
| Dopamine depleter | Reserpine, Tetrabenazine |
| Dopamine synthesis blocker | Methyldopa |

**Table B. Diseases with risk of secondary or atypical Parkinsonism**

| **Diseases** | **ICD-9-CM** |
| --- | --- |
| Stroke | 430-438/A290-A294,A299 |
| Dementia | 290,331.0, 331.2/A210 |
| Meningitis, encephalities | 00321,﻿0065,﻿0130-3,﻿0136,﻿0360-1,﻿0460-3,﻿047,﻿0490-1,0520,﻿0530,﻿0543,﻿05472,﻿0550,﻿05601,﻿062-4,﻿0721-2,﻿09041-2,﻿0941-2,﻿09481-2,﻿09487,﻿09882,﻿10081,﻿11283,﻿1142,﻿11501,﻿11511,﻿11591,﻿1300,﻿1390,﻿320-6 |
| Head injury | 800-1, 803-4, 850-4 |
| Hydrocephalus | 742.3, 741.0, 331.3-4 |
| Brain tumor | 191, 192.0-1, 192.8-9, 194.3-4, 198.3, 237.0-1, 237.5-6, 237.9, 239.6-7, 2250, 2252, ﻿2273-4, ﻿22802 |
| Congenital or hereditary disorders | 2750-1,﻿ 3334,﻿ 334, ﻿740 |
| Hypoxic encephalopathy | 348.1, 997.01, 639.8, 669.4, 768.7, 779.2 |

**Table C. Potential confounders**

| **Diseases** | **ICD-9-CM** |
| --- | --- |
| Hypertension (HTN) | 401-405 / A260,A269 |
| Diabetes (DM) | 250/ A181 |
| Hyperlipidemia | 272/ A182 |
| Coronary artery disease | 410-414 |

**Table D. Primary clinical outcomes**

| **Diseases** | **ICD-9-CM** |
| --- | --- |
| Accidental injury ^a^ | head injuries: 800–804, 850–854 and 959.01, A470,A490-1  bone fractures and dislocations: 805–808, 810–828 and 831–839  burns: 940–949  injuries to the spinal cord, plexus and nerves: 767.4, 767.6 and 952–957  superficial injuries and contusions: 910–924 |
| Dementia | 290,﻿331.0,331.2,A210 |
| Aspiration pneumonia | 482, but exclude 482.84 |

e-Reference:

^a^. Wang HC, Lin CC, Lau CI, Chang A, Sung FC, Kao CH. Risk of accidental injuries amongst Parkinson disease patients. Eur J Neurol. 2014 Jun;21(6):907-13.

**Table E. Dose equivalent of anti-depressants to fluoxetine (fluoxetine=1)**

| class |  | dose equivalent to fluoxetine (fluoxetine=1) |
| --- | --- | --- |
| tricyclic antedepressant (TCA) | Imipramine/ Imipramine oxide | 1/3.43 ^a^ |
|  | Clomipramine | 1/2.90 ^a^ |
|  | Amitriptyline | 1/3.06 ^a^ |
|  | Maprotiline | 1/2.95 ^a^ |
|  | Doxepin | 1/3.5 ^a^ |
|  | DOTHIEPIN | 1/3.87 ^a^ |
| selective Serotonin reuptake inhibitor (SSRI) | Fluoxetine | 1 ^a^ |
|  | Sertraline | 1/2.46 ^a^ |
|  | Paroxetine | 1.18 ^a^ |
|  | Fluvoxamine | 1/3.58 ^a^ |
|  | Citalopram | 1 |
|  | Escitalopram | 2.22 ^a^ |
| Serotonin/Norepinephrine reuptake inhibitor (SNRI) | Venlafaxine | 1/3.74 ^a^ |
|  | Duloxetine | 1/1.5 |
|  | Trazodone | 1/10 ^a^ |
| Norepinephrine-dopamine reuptake inhibitors (NDRI) | Bupropion | 1/8.71 ^a^ |
| Noradrenergic and specific serotonergic antidepressant (NaSSA) | Mirtazapine | 1/1.27 ^a^ |

e-Reference:

^a^. Hayasaka Y, Purgato M, Magni LR, Ogawa Y, Takeshima N, Cipriani A, Barbui C, Leucht S, Furukawa TA. Dose equivalents of antidepressants: Evidence-based recommendations from randomized controlled trials. J Affect Disord. 2015 Jul 15;180:179-84.
